# Hypnotism Medically Considered

**Published:** 1890-03-01

**Authors:** 


### The Hospital

A Weekly Journal of

**Science, Medicine, Nursing, and Philanthropy.**

*For Hospitals, Asylums, Medical Practitioners, Students, Nurses, Families, Parishes, Congregations, and the Charitable Public.*

Vol. VII.—No. 179.] [March 1, 1890.

### Hypnotism Medically Considered.

Doctors as a class, and among themselves, have a laudable hatred of humbug. Neither Carlyle nor even Teufelsdröckh himself had a more unsparing tongue for all kinds of wordy make-believe. When he addresses the public a medical man may indulge in a little rhetoric, and give occasional rein to his fancy, like any other mortal. But when he speaks to persons of like training and occupation with himself he is as hard down upon the bed-rock of material fact as he can possibly be. If materialism by itself could guide men unerringly in the ways of pure truth, honest and capable doctors would be the greatest philosophers alive. But what profession, having regard to its numbers, has ever shown so much diversity of opinion as the medical? We are thence entitled to conclude that even for medicine itself there might be some advantage if less materialistic methods of thought and action could be made to prevail.

Those members of the profession who have candid and open minds have seen with deep satisfaction a medical work on Hypnotism received with civility by the profession, and judged with fairness. Twenty years ago the author of such a work would probably have been denounced as a quack and a charlatan. It does not follow, of course, that because open-minded doctors have given impartial consideration to the facts brought forward about Hypnotism, therefore Hypnotism itself is well understood, and generally accepted as a valuable addition to therapeutic resources; but it does follow that the subject is likely to receive the winnowing of competent criticism, and that the grain which is in it, if any, is practically sure to be preserved and made use of.

Dr. Lloyd Tuckey is the English apostle of Hypnotism, and he gives a very good account of his Evangel in the *Medical Annual* for 1890, which has just been issued. Hypnotism is said to have been built up on the ruins of mesmerism and animal magnetism. That is a pretty figure of speech, which must not be taken for more than it is worth. To Mr. James Braid, a Manchester surgeon of forty years ago, belongs the merit of having invented the term, and of having put into practice those peculiar methods of treatment which he ranged under it. His method seems to have died with him so far as English practitioners were concerned. It will surprise some people—medical men as well as others—to learn that Dr. Liébeault, of Nancy, practised hypnotism in a public dispensary thirty years ago, and published the results of his experience in 1866. To Liébeault is due the honour of discovering the value of "suggestion" in the hypnotic state. Braid seems to have overlooked the very circumstance which constitutes the chief part of modern hypnotism. After more than twenty years of hypnotic work at Nancy on the part of Liébeault, Bernheim, Professor of Medicine at Nancy, entered upon an investigation of the subject, and, more recently, M. Charcot has taken it up with all his characteristic enthusiasm.

What, then, is Hypnotism? It is the putting of a person into a kind of sleep by the exercise of the nervous influence of another person. Is it possible for one person to put another into a real and actual state of sleep of this kind? Most certainly! Those who have seen it done repeatedly, as the writer has, never think of doubting it. This part of the treatment, therefore, is as much a reality and a fact as the reality and fact of sleep produced by any other means, though the "sleep" may differ in intensity. And, indeed, there is no more reason in the nature of things why the drinking of a solution of twenty grains of chloral hydrate or the inhaling of an ounce or two of chloroform should put a man to sleep than why nerve influence should fail to do so. Surely no modern physiologist will deny the actuality of nerve influence. If an eloquent preacher or a great tragedian can make an unintellectual and soulless London clerk in a top back gallery weep or laugh at his pleasure, it cannot be considered a strange thing if another man of equal sympathy and nerve force with the preacher should be able to induce in a person sitting close to him a condition of cerebral quiescence and insensibility. The sleep of the hypnotised patient is a nervous phenomenon, which may be classed with the laughter and the tears produced by the orator and the tragedian.

So far then we are dealing with facts that are universally admitted. It may be said that the hypnotic state is only a kind of waking sleep; and if dreams and waking sleeps had been previously unknown in human history, this objection would have deserved serious consideration. But admitting that hypnotic sleep is real, in what way can "suggestion" exert any actual and permanent influence either of a curative or any other kind on the hypnotised person? That is the first question asked by most people, doctors included. But such a question at such a time is entirely opposed to every method of modern science. The real question to ask is not, how can hypnotic suggestion effect a cure, but, does hypnotic suggestion ever effect a cure—of the drink-craving for example? Can a sufficient number of actual cures be brought to the cognizance of science on which to base an induction? If they can, then not only must we accept the facts if we are loyal to science, but we must use the treatment whether we can give any explanation of it or not. The very first business, surely, of all science is the gathering of facts; and the second is the using of them if they be of the least service to mankind. Their rational explanation comes at a later stage.

Experience, the experience of particular individuals, offers, we think, at least some hints which point towards an explanation of the *modus operandi* of suggestion. The writer remembers with remarkable clearness the circumstances of his second professional examination. He had read hard for many weeks, continuing his reading into the small hours of the morning. A condition of sleeplessness and brain irritability had thus been induced. The night before his *viva voce* was spent in complete wakefulness. The questions of the examiners, so far as they referred to familiar subjects, were answered with eager rapidity. But those questions to which answers were not ready seemed to burn into the brain like hot points of steel. They were never forgotten. They remain to this day; and to this day the smallest effort of the imagination can reproduce apparently the very sensations that were then experienced, and in the same part of the brain. The moment the examination was over books were consulted, and the subjects on which ignorance had been disclosed were studied afresh. One reading in that exalted state of the mind was abundantly sufficient. The knowledge gained under that influence has never been lost. Even now the reading of such subjects, or the reference to them by any other person, brings back in some degree the old unreasoning fears, the old mortification, and the old shame. Perhaps it should be stated that the examination was successfully passed. A peculiarity that then struck the writer, and which still remains with him, was the moral factor which entered into and took its share of the cerebral activity. He felt that he ought to have known the answers to all the questions put to him, although that of course was impossible; and he still feels as if it would be a disgrace if he did not know them now. And, as a matter of fact, he does know the answers to those particular questions which he then failed to answer, though a good many of those which he then answered readily he could not answer now. Is there not here a double indication which may help to elucidate the efficacy of hypnotic suggestion? There is, first, the fact that things impressed upon the brain when it is in a state of exalted sensibility are long remembered; and also the equally striking fact that a duty authoritatively affirmed to a brain in that condition, and acknowledged by it to be a duty, retains a wonderful tenacity of authority over the person when he has returned to his normal state. But whatever may be thought of the explanatory value of these facts, there can be no doubt that the real question at issue is not, Can hypnotism be scientifically explained, but can it be proved to be of real value to the conscientious medical practitioner?

